# Long-range regulation is a major driving force in maintaining genome integrity

**DOI:** 10.1186/1471-2148-9-203

**Published:** 2009-08-15

**Authors:** Emmanuel Mongin, Ken Dewar, Mathieu Blanchette

**Affiliations:** 1 McGill Centre for Bioinformatics, McGill University, Montreal, Canada; 2 Research Institute of McGill University Health Centre, McGill University and Genome Quebec Innovation Centre, Montreal, Canada; 3 Departments of Human Genetics and Experimental Medicine, McGill University, Montreal, Canada; 4 School of Computer Science, McGill University, Montreal, Canada

## Abstract

**Background:**

The availability of newly sequenced vertebrate genomes, along with more efficient and accurate alignment algorithms, have enabled the expansion of the field of comparative genomics. Large-scale genome rearrangement events modify the order of genes and non-coding conserved regions on chromosomes. While certain large genomic regions have remained intact over much of vertebrate evolution, others appear to be hotspots for genomic breakpoints. The cause of the non-uniformity of breakpoints that occurred during vertebrate evolution is poorly understood.

**Results:**

We describe a machine learning method to distinguish genomic regions where breakpoints would be expected to have deleterious effects (called breakpoint-refractory regions) from those where they are expected to be neutral (called breakpoint-susceptible regions). Our predictor is trained using breakpoints that took place along the human lineage since amniote divergence. Based on our predictions, refractory and susceptible regions have very distinctive features. Refractory regions are significantly enriched for conserved non-coding elements as well as for genes involved in development, whereas susceptible regions are enriched for housekeeping genes, likely to have simpler transcriptional regulation.

**Conclusion:**

We postulate that long-range transcriptional regulation strongly influences chromosome break fixation. In many regions, the fitness cost of altering the spatial association between long-range regulatory regions and their target genes may be so high that rearrangements are not allowed. Consequently, only a limited, identifiable fraction of the genome is susceptible to genome rearrangements.

## Background

Genomes evolve through a series of local mutations as well as larger-scale genome rearrangements (such as inversions, translocations and duplications) where one or more chromosomes break in one or more locations (called breakpoints) and fragments are reorganized. Just like for point mutations, the likelihood that a particular rearrangement becomes fixed in the population depends (in part) on the fitness of the mutated individual [1]. In comparative genomics, the comparison of gene orders in different species (i.e. of those rearrangements that have become fixed in their respective population) sheds light on genome evolution [2,3] and phylogenetics [4-6].

In 1984, Nadeau and Taylor published a paper where breakpoints of genome rearrangements (chiefly inversions and translocations) between human and mouse are modeled as occurring randomly and uniformly in the genome [7], hypothesis later supported by Sankoff *and *Trinh [8]. This model relies on the implicit assumption that most breaks of synteny (disruption of the order of markers, genes or regulatory elements, along a chromosome caused by genome rearrangements) do not have significant functional implications. However the availability of more genomes to undertake comparative genomic studies and new algorithms to identify breakpoints increased both the resolution and the completeness of the analysis. This led to a model where rearrangements breaks do not occur uniformly but instead where some regions, termed as 'evolutionary hotspots', are more prone to breakage, resulting in a high level of breakpoint reuse [2,3]. Although it is now generally accepted that evolutionary breakpoints (i.e. rearrangement breakpoints that became fixed in a particular population) are not uniformly distributed on the human genome, the reasons why some regions tend to fix chromosomal rearrangements more than others still remains unclear and to date, no satisfactory explanation has yet been given at the whole genome level. Long-range regulation has been hypothesized to be one of the elements that favor conservation of synteny in certain regions of the genome [9]. Studies focusing on specific vertebrate regions, such as the Hox cluster [10] or the Shh locus [11], where a strong selective pressure is obviously at work, illustrated the notion that regulatory regions surrounding those loci could induce evolutionary constraints that maintain the integrity of the genome. Kikuta *et al*. [12] and Engstrom *et al*. [13] established that some regions are under the influence of what they designated as genomic regulatory blocks (GRBs), which control the expression of developmental genes over a large genomic region, and showed that the synteny around those GRBs is maintained. They suggested that the underlying mechanism that maintains the chromosomal structure is the regulatory action of one element on many different genes. To date no genome wide analysis has been undertaken to uncover the different susceptibility of the human genome to breakpoints. Such information is crucial to clarify on the forces preventing breakpoints from being fixed in evolution.

In this paper, we propose a new approach to estimate the susceptibility of regions of the human genome to tolerate breakpoints. Our method is trained to recognize these regions based on the presence of coding, conserved non-coding elements (assumed to be enriched for regulatory regions) and their putative interactions. We were able to define two types of regions: those that are prone to accept evolutionary breakpoints and those that are refractory to breakpoints. The analysis of those regions uncovers features that shed some light on the underlying mechanisms of selection against rearrangements. This suggests that long-range regulation is a major driving force in maintaining genome integrity.

## Results and discussion

### Results

#### Synteny mapping

In this analysis, we study breakpoints that occurred along the human lineage since the metatheria divergence (eutherians vs marsupials split). These breakpoints can be identified through the comparison of the human genome to that of a marsupial (here, opossum [14]), and an outgroup, chicken [15]. Identifying breakpoints requires the detection of unique, conserved markers, present in each of the species studied. Based on whole genome 'liftover chains' pairwise alignments [16] (human/opossum and human/chicken) which are a hierarchical collection of sequences of gapless aligned blocks, we mapped human markers to opossum and chicken. Markers are of two types; non-coding conserved regions which are considered enriched for regulatory elements and coding regions. 116 331 markers were identified, each present exactly once in human, opossum, and chicken. We call these *amniote *markers. We also identified 93 802 *metatherian *markers, conserved between human and opossum, but absent in chicken. Metatherian markers will not be used to define breakpoints (because no outgroup is available to determine their ancestral status), but will later be taken into consideration in our prediction of break-prone regions.

A breakpoint between human and opossum (resp. chicken) is defined as a pair of amniote markers that are adjacent in human but not opossum (resp. chicken). Because establishing the orthology of human, opossum, and chicken markers is error-prone, we deliberately removed from further consideration 383 markers that are flanked by breakpoints on both sides in either the human/opossum or human/chicken comparisons. Although some of these breakpoints may be real, we argue that most of them are likely due to incorrect genome assembly or orthology mapping. This reduced set of markers was then used to define a set of 845 reliable human/opossum and 1546 human/chicken breakpoints. The intersection of the human/opossum and human/chicken breakpoints, which corresponds to 412 breakpoints that took place along the human lineage since the divergence of metatherians, is called the set of *human breakpoints *and is the focus of our study in the rest of this paper.

As expected, breakpoints within protein-coding genes are rare, forming only 2.7% of human/opossum breakpoints and 3.4% of human/chicken breakpoints. Many of these intragenic breakpoints are likely to be the result of incorrect gene annotation. For example, annotated genes such as MPP4 are made of multiple spliced variants gathered together in one gene, but it could very well correspond to two independent transcriptional units. Because of this, the few human breakpoints occurring within annotated genes were pushed to the left side of the gene. Annotated genes now being free of breakpoints, all markers within them (whether they are coding or not) were collapsed into a single meta-marker called a coding marker. The number of markers of each type is given in Table 1.

**Table 1:** Number of markers for each type and conservation level.

Marker type	metatheria	amniote	total
coding	4335	9951	14286
non-coding	46914	26217	73131

Coding markers are genes as annotated in EnsEMBL and non-coding markers are non-coding conserved regions (taken from the UCSC 28-way alignment). A human marker (coding or non-coding) conserved only at between human and opossum is labeled as metatheria marker. A human marker conserved both with opossum and chicken is labelled amniote marker.

What factors determine the likelihood that a particular rearrangement becomes fixed in a population? The main factor is likely to be the difference of fitness between individuals with and without the rearrangement. While a given rearrangement is rarely going to be beneficial to the affected individual, it may very well be detrimental. Three situations may be particularly deleterious: (i) when a breakpoint occurs within a gene, (ii) when a breakpoint occurs between a gene and a cis-regulatory element for that gene, thus separating this gene from its regulator, and (iii) when a rearrangement brings a regulatory element in the vicinity of a gene leading to its mis-regulation. See also [17] for population genetics consideration. To predict the potential effect of a breakpoint at a given genomic position, it is useful to look at the context within which the breakpoint happened (the ancestral state), rather than the result of that rearrangement (the derived state). Of course, we do not have access to the exact ancestral state surrounding each breakpoint.

However, because breakpoints are rare and, for the most part, separated by fairly large genomic distances [2], the genome of the closest extant species outside the lineage on which the rearrangement occurred provides a good approximation of that local ancestral state. In our case, since we focus on breakpoints on the human lineage, the ancestral state can be approximated using the opossum local context. Moreover, we only consider syntenic blocks consisting of at least of two markers, which excludes most micro rearrangements. This approximation does not take in account events that may have occurred in the same region on the opossum branch after divergence, but since the goal of this study does not require a high level of precision, this method is, in our point of view, sufficient. Moreover, trying to computationally infer the real ancestral state could lead to errors that may add noise and not improve the prediction.

**Figure 1:**
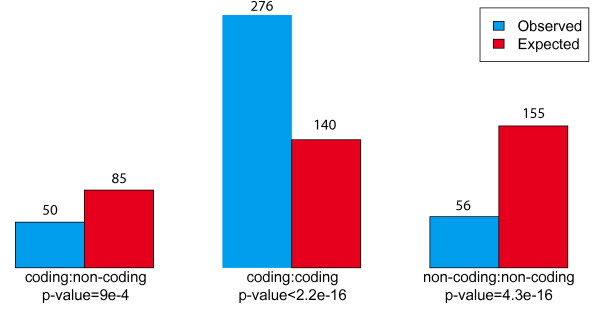
**Breakpoints and surrounding markers**. Observed (blue) and expected (red) number of breakpoints depending on the types of flanking ancestral markers. The expected number of breakpoints was calculated based on the total size of inter-marker regions of each type. P-values were calculated with a chi-square test.

Figure 1 shows that there is a strong enrichment for breakpoints occurring between two coding markers and a strong depletion of breakpoints flanked by one or two non-coding markers, supporting the hypothesis that regulator/target-gene relations severely constrain breakpoint fixation. Following this observation, we aimed at understanding better what properties of a given genomic region increases or decreases its likelihood of being involved in a breakpoint that would become fixed in the population, and to train a classifier to predict break-prone inter-marker regions based on their context. Our data set thus consisted of 383 positive examples (the human-lineage breakpoints, considered in their ancestral (opossum) context), and 35 586 negative examples (inter-marker regions without breakpoints). It should be noted that inter-marker regions over 1 Mb in opossum an human have been removed from consideration because breakpoints couldn't be located sufficiently precisely.

#### Features used for breakpoint prediction

**Figure 2:**
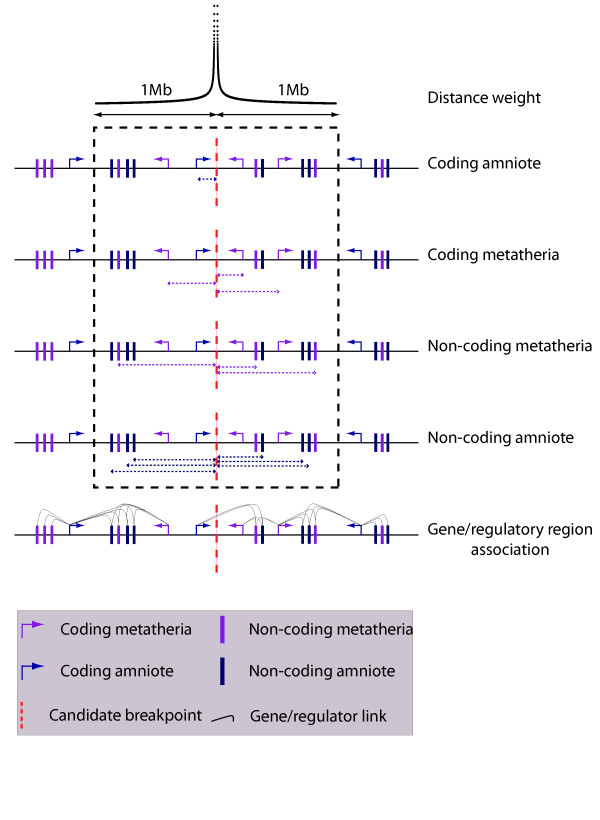
**Feature selections**. Diagram representing the different types of features used by our predictor. For the local density of functional elements, the categories are coding amniote, non-coding amniote, coding metatheria, non-coding methateria within a 2 Mb window. For each of these categories, the distance from the candidate breakpoint to the center of inter-marker region considered is taken in account and various weighting factors termed *α *are applied. The effect of an *α *factor of 2 is presented at the top of the figure. In that case markers close to the breakpoint have a high weight whereas distant markers only bring a small contribution. The functional association between regulatory regions and genes is a different category of features. Each non-coding element is associated with a set of genes depending on the value of the *β *factor, which determines how far the association between non-coding and coding regions will be considered (*β *= 2 is illustrated). For a given inter-marker region, the predictor will test if the region overlaps such associations. In total, 29 features are considered.

Two types of features were used for breakpoint prediction (see Figure 2); the local density of functional elements and the association between non-coding putative regulatory regions and genes. The local density of each type of functional elements (coding-metatherian, coding-aminote, noncoding-metatherian, noncoding-amniote) is measured as a weighted count of such elements in a 2 Mb-window centered on the region of interest. The weight of an element decreases as a function of its distance from the center of the window, as *w*(*d*) = 1/log(*d*)_*α*_. Choosing *α *= 0 gives the same weight to all elements within the window, whereas a high *α *factor (*α *= 3) gives a much higher weight to elements close to the center.

The other type of features considered describes the relationship between non-coding conserved regions (considered in this study as enriched for cis-regulatory elements) and their putative target coding regions. This relationship is described as a function of a parameter *β*. With *β *= 1, each non-coding marker is linked to the gene with the closest transcription start site (up to a maximal distance of 1 Mb). For *β *> 1, each non-coding marker is linked to its closest gene and to all other genes located within at most *β *times the distance to the closest gene. Consequently, the higher the *β*, the more genes are linked to a single non-coding region. The 'association' feature of a given inter-marker region is then defined by the number of such association that would be broken by a break in that region. These features have been chosen to test the hypothesis that long-range regulation may be a factor in maintaining the integrity of the genome. Under that hypothesis, breakpoints would be expected to occur where no (or few) regulator-target gene connections are broken.

#### Removing inter-marker distance bias

Unsurprisingly, the length of an inter-marker region is strongly correlated with its likelihood to contain a breakpoint (logistic regression analysis, p-value = 4.5e-6). This confounding factor needs to be factored out before more interesting predictive features can be teased out. To this end, we fitted the breakpoint/no-breakpoint binary data using a linear regression based on inter-marker fragment length (Breakpoint(r) ~ *a*·Length(r) + b) and obtained the residuals of the regression (Residual(*r*) = (Breakpoint(*r*) - *b*)/*a*). Large inter-marker regions with no breakpoint produce large negative residuals, while small inter-marker regions with a breakpoint produce large positive residuals. It is these residuals, which should be considered as fragment labels normalized for fragment lengths, that are used as target values for the predictors that follow.

#### Breakpoint predictors

**Figure 3:**
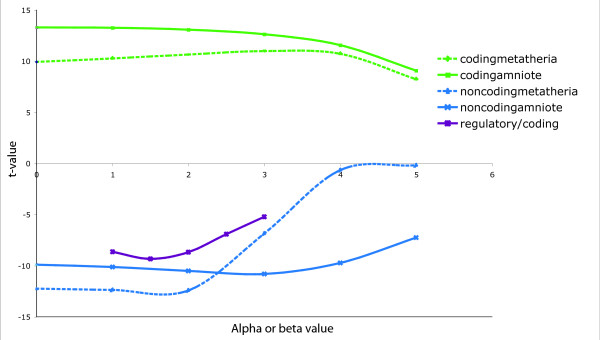
**Effect of single feature on the prediction**. t-value obtained for the linear regression of length-normalized breakpoint data for the different types of local density features (coding amniote, noncoding amniote, coding metatheria, noncoding metatheria), for different values of *α*. On the same graph are represented the t-values of the regression against the presence of association between conserved non-coding elements and genes, for different values of *β*.

We first tested the predictive value of each individual feature. Then, the best combination of features was selected with a forward feature selection procedure. Each feature was first tested independently to examine its ability to predict breakpoints, measured by the t-value of the linear regression of the length-normalized breakpoint data against that feature. A graph showing both the effect for local density features and relationship between coding and non-coding features (for different *β *values) is presented in Figure 3. A large negative t-value represents a negative correlation of the feature with the presence of breakpoints, whereas a large positive t-value indicates that the presence of this feature is favorable to breakpoints. We observe that a high local density of coding elements (both metatherian and amniote) is associated to an increased likelihood of breakpoints, corroborating our previous observation that breakpoints occur more often than expected between coding markers. This is in accordance with observations showing that the synteny of conserved non-coding elements within gene deserts is usually well conserved [18]. Interestingly the density of more ancient genes (amniote) is more strongly associated to breakpoints than that of more recent ones. Indeed, as we will see bellow, most housekeeping genes are shared among amniotes and they are also associated to such breaks. In addition, we note that the value of the locality parameter *α *has little impact on the fit, suggesting that the best predictive features would be a more complex function of the distance. On the other hand, non-coding markers (or putative regulatory regions) are negative predictors for breakpoints. Interestingly, although non-coding amniote and metatherian densities are strong negative breakpoint predictors when *α *is small, only the non-coding amniote density remain predictive for large values of *α*, indicating that breakpoints in the immediate proximity of such ancient regulatory elements are quite rare, but that breakpoints near more recent non-coding are less deleterious. Features modeling the association between coding and non-coding markers have a negative t-value, which means that breakpoints are less likely to become fixed in the population if it breaks such association. Finally, the best t-value obtained is for *β *= 1.5, indicating that the regulator/target gene relation often is not limited to a non-coding region and its (single) closest gene.

#### Predictor training and cross-validation

**Figure 4:**
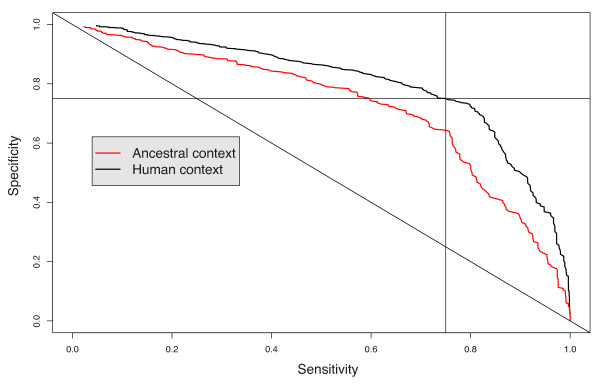
**Specificity/sensitivity curve for breakpoint predciction**. Specificity/sensitivity curves are presented for both predictions (ancestral context or the human (derived) context).

A multiple linear regression breakpoint predictor was built using a forward feature selection procedure, whereby we iteratively add to the predictor the feature that yields the largest accuracy improvement, until no further addition is beneficial. To test the performance of each intermediate and final predictor, we performed a four-fold cross-validation. Instead of measuring the accuracy of our predictors in terms of the fraction of inter-marker region correctly predicted to have a breakpoint, the sensitivity and specificity of each predictor was assessed in terms of the total fraction of the genome predicted to be breakpoint-sensitive. Table 2 reports the result of the feature selection procedure. Choosing the appropriate prediction score threshold, the predictor identifies 35.5% of the genome as breakpoint-prone, and these regions indeed contain more than 75% of the actual breakpoints, more than twice the expected accuracy of a random predictor. After assessment of its performance, the predictor was trained on the whole data set (using opossum as an approximation to the ancestral context) and applied to the prediction of breakpoints in the human context. Surprisingly, this predictor outperforms the original one at predicting past human breakpoints succeeding at capturing 75% of breakpoints in break prone regions covering only 27% of inter-marker regions (see Figure 4), indicating that either the opossum genome is not a very good approximation to the ancestral context, or that the derived state matters as much as the ancestral state to predict breakpoint fixation.

**Table 2:** Effect of each selected feature on the prediction.

Feature	Estimate	Std. Error	t value	p-value	specificity
CodingAmniote, *α *= 0	0.0030645	0.0005186	5.910	3.47e-09	0.473
AssociationBreaks, *β *= 2	-0.0150509	0.0022701	-6.630	3.42e-11	0.572
CodingMetatheria, *α *= 2	1.3915142	0.2395547	5.809	6.36e-09	0.611
NonCodingMetatheria, *α *= 3	-0.0338532	0.1649918	-0.205	0.83743	0.625
CodingMetatheria, *α *= 0	-0.0079558	0.0014829	-5.365	8.15e-08	0.629
NonCodingMetatheria, *α *= 2	-0.0154798	0.0132948	-1.164	0.24429	0.630
CodingAmniote, *α *= 3	-4.6993169	0.9579974	-4.905	9.38e-07	0.634
AssociationBreaks, *β *= 3	0.0069784	0.0024624	2.834	0.00460	0.645
NonCodingMetatheria, *α *= 4	0.0302276	0.1104565	0.274	0.78435	0.645

Features are listed in the order in which they were selected by the forward feature selection procedure. The coefficient estimate, standard error, t-value, and p-value reported are those obtained for the linear regression with all 9 features. The specificity reported is that of the predictor built using the features starting from the 1st row down to the current row. The specificity is calculated for a sensitivity of 0.75. For example a specificity of 0.645 means that 75% of breakpoints are comprised within 35.5% of the total length of inter-marker regions used for the analysis.

**Figure 5:**
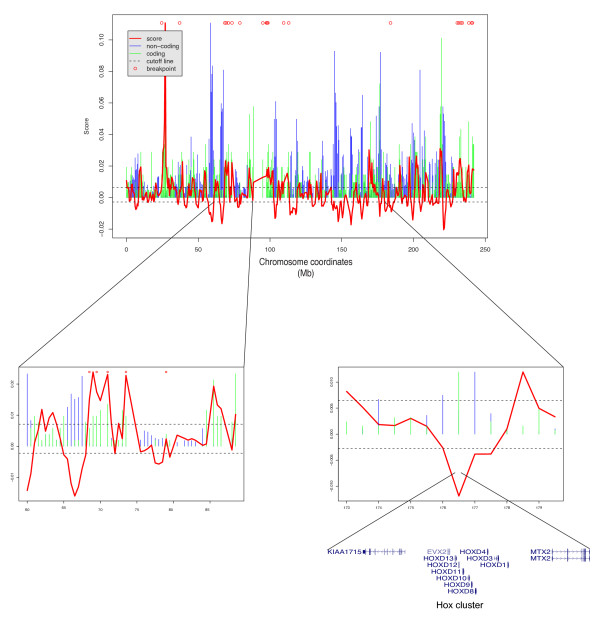
**Prediction scores on chromosome 2**. Normalized number of non-coding and coding elements are respectively represented with blue and green bars. Scores are represented by a red continuous line. The bottom right part of the figure show the region corresponding to chromosome 2 Hox cluster. The bottom left part shows a region of chromosome 2 with a high density of breakpoints.

Figure 5 shows the breakpoint susceptibility profile of human chromosome 2, using a 500 kb sliding window. This score is strongly positively correlated with the number of coding regions and negatively correlated with the number of non-coding conserved regions. However, some regions, such as the Hox D cluster on chromosome 2, a gene rich region but known to contain several evolutionary conserved non-coding regions, is also predicted with a low score. For all predictions, see Additional file 1.

#### A limited fraction of the genome can tolerate breakpoints

The predictor was applied to the complete human genome to identify regions that are more likely to tolerate breakpoints (see Supplemental Material). We divided the genome into two sets of regions: *susceptible *regions are those predisposed to rearrangement (regions with score above 0.0065, covering 30% of the non-genic euchromatic genome), and *refractory *regions, referring to those that are resistant to rearrangements (regions with score below -0.0028, covering 30% of the non-genic euchromatic genome). About 73% of human breakpoints are comprised in susceptible regions (a 2.3-fold enrichment), while only 7% are found within refractory regions (a 4.3-fold depletion). Most of the breakpoints are then contained in a limited fraction of the genome. This clearly shows that breakpoint fixation is not happening randomly and uniformly across the genome and that regions that are more likely to be broken can be predicted. Moreover if we consider that breakpoints almost never occur within genic regions or within conserved regions, we obtain that more than 73% of the human breakpoints are located in about 20% of the genomic regions considered. This observation complements the theory of evolutionary hotspot described by Pevzner *et al*. [3]. We then used this classification to uncover additional properties of each type of regions.

#### Susceptible and refractory regions have different characteristics

Susceptible and refractory regions differ in a number of aspects.

1. **Refractory regions are strongly enriched for non-coding markers, and susceptible regions for coding markers**

The ratio of coding to non-coding markers is significantly higher in susceptible regions than in refractory regions (p-value < 2^-16^, Fisher test, see Table 3). This result meets observations made by Murphy *et al*., showing that there is a significant increase of gene density in breakpoint regions [19].

**Table 3:** Properties of refractory and susceptible regions.

	Refractory regions	Susceptible regions	Refr./Susc. Ratio
Coding markers	1484	7423	0.20
Noncoding markers	41947	5576	7.5

Specific genes	374	1636	0.23
Ubiquitous genes	194	1485	0.13

Gene deserts	142	5	35.5

2. **Refractory regions are enriched for Trans/Dev genes**

**Figure 6:**
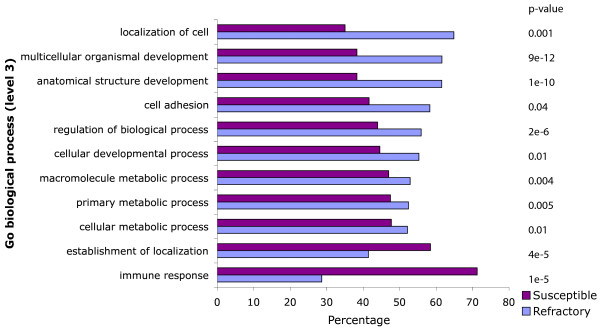
**GO categories enrichment and depletion in susceptible and refractory regions**. Over-and under-represented GO categories (biological process, level 3 only) for genes localized within susceptible and refractory regions. The adjusted p-values were obtained with a two-sided Fisher test using the Fatigo function from the Babelomics platform.

A Gene Ontology analysis (performed on the "biological process" classification with the Babelomics platform [20]) reveals that refractory regions are strongly enriched for genes involved in development, such as anatomical structure development (p-value 1 × 10^-10^), multicellular organismal development (p-value 9 × 10^-12^) and regulation of biological process (p-value 2 × 10^-6^) (see Figure 6). This confirms the observation made that developmental genes are enriched in syntenic regions [13]. Interestingly, susceptible regions are enriched for genes involved in immune response. These genes must be extremely adaptive and genes such as immunoglobulin are under intense gene diversification processes such as gene conversion, somatic hypermutation and class switch recombination [21]. It is then not surprising to predict higher rearrangement rates in regions involved in immunity which are under strong positive selection pressure. However, this may also be an artifact caused by the intense duplication history of some of these genes, which makes them more susceptible to misalignment.

3. **Refractory regions are enriched for tissue specific genes.**

We used the GNF Expression Atlas 2 [22] to classify the human genes based on their expression in 79 human tissues and cell types. The dataset contains expression measurements for 14 614 distinct Ensembl genes. Each gene was classified according to the number of tissues in which it is expressed [23]. A gene is considered expressed if the detected expression level is above a certain threshold. Using this classification method, two gene sets were created. The set of 'specific' genes consists of all genes expressed in at most 5 tissues and contains 3 235 genes (see Methods). The set of 'ubiquitous' genes contains 2 520 genes expressed in more than 70 tissues. The remaining genes expressed in 6 to 69 tissues where not used for this analysis. The ratio between the number of specific genes and ubiquitous genes is clearly imbalanced between refractory and susceptible regions.

Refractory regions are clearly enriched for specific genes compared to susceptible regions (two-sided Fisher-test, p-value 3 × 10^-9^, see Table 3).

4. **Most gene deserts lie in refractory regions.**

**Figure 7:**
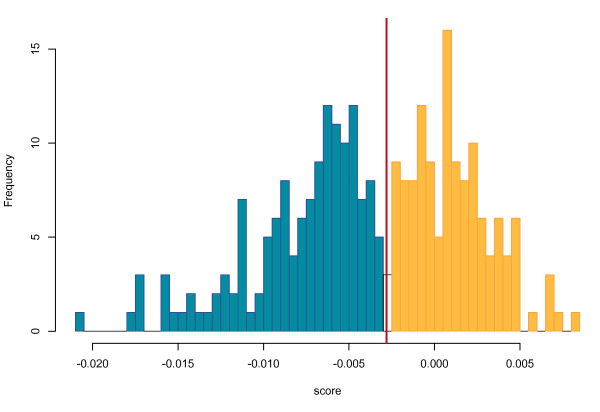
**Distribution of the average prediction score of gene deserts**. The red line corresponds to the threshold below which a region is considered refractory. The distribution is clearly bimodal, as highlighted by the coloring scheme.

About 25% of human genome is composed of gene deserts, which are defined as long inter-genic regions [24]. In this work, we define a gene desert as a genomic region of more than 1 Mb without protein coding genes. The human genome contains 270 gene deserts, of which 142 fall within refractory regions (based on their average score) but only 5 within susceptible regions (the 123 others are located in regions that are neither predicted as refractory nor susceptible). This result agrees with our previous observation that most breakpoints avoid non-coding conserved regions and is consistent with previous studies stating that most gene deserts are not broken by evolutionary breakpoints [18]. The predicted scores in gene deserts follow a bimodal distribution, as shown Figure 7. From this distribution, we can distinguish two types of gene deserts: (i) those whose score is under the refractory threshold, where evolutionary breakpoints are not likely to happen and (ii) those over this threshold. Interestingly, this dichotomy of gene deserts for susceptibility to breakpoints is somewhat similar to observation made by Ovcharenko et *al *[18], who noted that gene deserts can be separated into two kinds: 'stable' and 'variable'. Stable and variable gene deserts are described with different properties: genes flanking stable gene deserts are enriched for transcriptional and developmental functions and are resistant to rearrangements. This meets our observations on susceptible and refractory regions.

5. **Susceptible regions are enriched for copy number variants**

Copy number variants (CNVs) are regions (1 Kb to 1 Mb) of the human genome whose copy number is polymorphic in the population. From the database of genomic variants [25], we retrieved the base pair coverage for copy number variations in susceptible and refractory regions. As the database contains various kinds of variation such as inversions, we selected only variations labelled as copyNumber. CNVs are significantly enriched in susceptible regions, compared to refractory region. 25.1% of base pairs in susceptible regions are covered by CNVs, whereas this is the case for only 19.6% of those in refractory regions (see Table 4). Regions with high coverage of CNVs are indeed regions of the genome where variations in size are potentially less detrimental. It is not surprising then to find an enrichment for CNVs in regions predicted as susceptible to breakpoints. This CNVs analysis is another independent confirmation of the validity of our predictor.

**Table 4:** Percentage of susceptible, neutral and refractory regions covered by rare, common fragile sites and CNVs.

	% covered by rare fragile sites	% covered by common fragile sites	% covered by CNVs
Susceptible regions	8.8	24.7	25.1
Neutral regions	4.5	22.3	22.3
Refractory regions	5.5	20.7	19.6

The statistical significance of the enrichment between refractory and susceptible regions for rare and common fragile sites as well as CNVs regions was assessed with a permutation test. The resulting p-values are < 10^-4^

6. **Susceptible regions are enriched for rare fragile sites**

Fragile sites are regions of the genome which appear as gaps or breaks on metaphase chromosome when exposed to inhibitor of DNA synthesis. Those regions are considered as 'unstable' part of the chromosome [26]. Fragile sites are further categorized depending of their frequency: rare fragile sites are present in a small proportion of individuals whereas common fragile sites are present in all individuals and are considered part of the chromosome structure [26,27]. We used 119 fragile sites (88 defined as common and 31 as rare) reported by Schwartz *et al*. [28].

Rare fragile sites are clearly enriched in susceptible region compared to neutral and refractory region, see Table 4. Those data meet observations already made by Ruiz-Herrera *et al*. [27] who showed a weak correlation between common fragile sites and evolutionary breakpoints and a more significant correlation between evolutionary breakpoints and rare fragile sites. We should point out the difference of resolution between the cytogenetic bands representing fragile sites (which are on average 7 Mb long) with our estimate of susceptible and refractory regions, which is more refined.

### Discussion

#### Breakpoints are bound to specific regions

In this study, we developed a predictor to define regions of the human genome that are likely to tolerate rearrangements. Using this predictor, we defined two classes of regions. Susceptible regions correspond to 30% of the intergenic genome and contain 73% of the breakpoints. Refractory regions correspond to 30% of the intergenic genome but contain only 7% of the breakpoints. Most breakpoints are then contained in a small portion of the genome. Considering that coding loci are also extremely refractory to breakpoints, only 20% of the human regions considered for the analysis are prone to rearrangements. This model – that breakpoints are concentrated in a small, identifiable fraction of the genome – complements the 'fragile breakage' model proposed by Pevzner and Tesler [2], which was developed as an alternative model to the random breakage theory introduced by Nadeau and Taylor [7].

#### Long-range regulation imposes functional constraints on the genomic structure

Regulatory regions and genes can be functionally associated over long stretches of DNA. Some regulatory regions have indeed been located as far away as 1 Mb away from their target genes (Shh long-range enhancer, for example [29]). Vavouri *et al*. also showed using duplicated conserved non-coding elements and paralogous genes that about half of non-coding elements are > 250 Kbp away from their target gene [30]. This long-range interaction associated with the complex relationship between regulatory regions and target genes (a gene can be targeted by many regulators and a regulator can target many different genes) establishes an important pressure to keep those regulators and target genes together. So, intuitively, the cost of breaking the physical relationship between long-range regulatory regions and their target genes may be so high that rearrangements are rarely fixed where such relationship is predominant (see also [31]). The analysis of susceptible and refractory regions, sheds light on the validity of this hypothesis.

#### Susceptible and refractory regions are functionally different

Refractory and susceptible regions have many distinguishing features: (i) Refractory regions are significantly enriched for putative regulatory regions and gene deserts; (ii) the ratio of housekeeping genes to cell type/tissue specific genes is higher in susceptible regions than in refractory regions; (iii) refractory regions are clearly enriched for genes involved in transcriptional regulation and developmental processes (trans/dev genes). Those distinct features show a functional dichotomy between susceptible and refractory regions, between regions that are involved in complex processes (e.g. transcriptional regulation of developmental genes) and regions enriched for housekeeping genes and depleted for non-coding conserved regions. This dichotomy is in our point of view a strong argument supporting the hypothesis that long-range regulation imposes constraints on the genomics structure. This confirms previous observations where synteny blocks overlap regulatory domains [13]. For example, transcription factor genes – enriched in refractory regions – are under complex regulation and can be expressed at different levels, at different times and in different tissues [32]. We also showed that copy number variants – an independent dataset – are enriched in susceptible regions in comparison to refractory region. If it does not bring any information on the cause of the instability, it however can be interpreted as the result of a reduced constraints on genome structure which could be due to decreased regulation complexity.

#### Reduced regulation complexity: cause or consequence of breakpoint susceptibility?

It has been shown that there is a significant overlap between evolutionary breakpoints and fragile site locations and that, even if no mechanistic role could be demonstrated, some fragile regions of the genome may be more likely to experience reorganization [27]. One may then wonder whether the relative regulatory simplicity observed in susceptible regions may actually be a consequence (rather than a cause) of the presence of fragile regions nearby. But although fragile regions are correlated with regions susceptible to breakpoints as shown by our data, they only represent a small fraction of susceptible regions, suggesting other mechanisms explaining those different levels of plasticity on the genome. If fragile regions may contribute to the fixation of breakpoints, it seems that the main mechanism preventing breakpoints is the crucial role that long-range regulation has on the fitness of the individuals.

#### Limitation of the model and further developments

The predictor was trained using the opossum genome as an approximation for the ancestral eutherian genome. Surprisingly the predictor performs better when using the current human genome (i.e. the derived genome), rather than the approximated ancestral genome, for predicting human breakpoints (see Figure 4). This outcome may be explained by the discrepancy in the quality of assembly and annotation between human and opossum. Nonetheless, we believe that using opossum as an approximation of the ancestral state is justified as the alternative, using a computationally predicted ancestral genome, may lead to a worse approximation because of reconstruction errors. Another observation to make from this discrepancy is that the derived state may be as suitable as the ancestral state to predict breakpoints. Indeed, the effect of a genome rearrangement is a combination of both the regulatory associations it disrupts (observable in the ancestral genome) and the new associations it creates (observable in the derived genome).

## Conclusion

We show in this study that the reason why some regions of the genome are not prone to rearrangement is that some of the genes they contain are under the influence of long-range regulators and the physical relationship between these elements cannot be broken without being detrimental to the fitness of the individual. Genes with simpler regulation, such as housekeeping genes, may be less affected by breakpoints in their surrounding. The consequence is that regions where rearrangements can be fixed and are not too detrimental correspond to regions that are enriched for genes with less complex regulation. In the light of these data, we confirm that the random breakage model is not the most appropriate and that only a limited fraction of the genome is susceptible to evolutionary rearrangements. The mapping of these regions, produced by our predictor, will be of importance for future genome evolution and function studies.

## Methods

### Marker identification

In order to undertake our analysis on the synteny of human putative regulatory regions and coding regions, we defined both datasets from publicly available data. As it is widely accepted that non-coding regions under selective pressure are enriched for regulatory regions, we selected the set of non-coding conserved regions from the human 28-way [33] alignment identified by PhastCons [34] and available on the UCSC genome browser [16]. Only regions longer than 50 bp and with a score over 400 (third quartile from the complete distribution of Phastcons elements) were considered. These regions were filtered out for ESTs, coding regions (exons), blastp hits and repeats using Ensembl annotations. Coding regions are defined from the set of human coding exons from the Ensembl version 49 [35]. When two exons overlap (which occurs in the case of splice variants), only the longest exon is considered. In the case of two overlapping genes (e.g, intronic gene), only the longest gene is taken into account. Through this process, we selected 216 300 exons (coding markers) and 112964 non-coding conserved regions (non-coding markers) to undertake the analysis.

### Ortholog mapping

Based on whole genome 'liftover chains' pairwise alignments (human/opossum and human/chicken) were retrieved from the UCSC genome browser [16]. Liftover chains are extracted from UCSC nets generated from blastZ alignments. Nets are a hierarchical collection of ordered aligned blocks and the mapping provided by this alignments is then unlikely to be spurious. Human (NCBI build 36.1) is used as the reference genome, and human conserved regions (coding and non-coding) are mapped using liftOver (forward mapping) to the chicken v2.1 draft assembly (WUSTL) and the opossum draft assembly (The Broad Institute, january 2006) (liftOver parameters: minMatch = 0.8 for opossum, minMatch = 0.7 for chicken). In order to only consider best reciprocal hits, the forward mapping results are mapped back to human (reverse mapping) using liftover (minMatch = 0.75 for opossum, minMatch = 0.65 for chicken). Markers lying on unknown chromosomes of the human genome or mapping to unknown chromosomes on one of the target genomes are discarded. Each marker (coding or non-coding) is then classified using its level of conservation. Markers conserved only between human and opossum are classified as metatherian. Those conserved between human and both chicken and opossum are classified as amniote markers (markers conserved only between human and chicken but not opossum were ignored).

### Synteny

A breakpoint between the human genome and the opossum genome (resp. the chicken genome) is defined as a pair of amniote markers (coding or non-coding) that are adjacent in human (disregarding possibly intervening metatherian markers) but not in opossum (resp. chicken). The only exception is that if the two markers are more than 1 Mb apart, the inter-marker region is disregarded, as in that case the resolution would be too low. This removes from consideration cases such as centromeres. As the exact breakpoint position cannot be determined, its localization is defined as the equidistant position between the two markers.

In order to undertake analyses at the gene level, exons are assembled into genes using the Ensembl annotation, and synteny breakages are ported at the gene level (placing the breakpoint on the left side of the gene. Marker classification (amniote and metatheria) is also ported from exons to genes. If at least 30% of the exons are labelled as amniote, the amniote annotation is ported to the gene. If at least 30% of the exons are labelled as metatheria (and less than 30% are labelled as amniote), the annotation metatheria is ported to the gene. Finally, those breaks retrieved on human were mapped to the opossum genome where the predictor training is undertaken.

In order to evaluate the significance of the enrichment of observed breakpoints depending of the flanking markers, we calculated the number of expected breakpoints based on the total size of inter-marker regions of each type using a chi-square test.

### Breakpoint prediction

Inter-marker regions were divided into two classes; syntenic regions and breakpoint regions. To train the predictor, the following information was used: local density of functional elements and association between putative regulatory regions and genes. A score summarizing the local density of elements within 1 Mb of the center of each inter-marker region was considered. The following elements were considered: the status of markers (coding or non-coding), their classification (metatheria or amniote), and their weighted distance. For a 2 Mb window *W *centered at genomic position *p*, feature scores were calculated as follows: , for *X *∈ {CodMet, NoncodMet, CodAmn, NoncodAmn} and *α *ranging from 0 to 5 (with increments of 1). Another feature considered is the connectivity between non-coding putative regulatory regions and genes. All non-coding conserved regions are associated to the gene with the closest transcription start site. In addition, a non-coding region is associated to a gene if the distance between them is at most *β *times more than the distance to the closest gene, for *β *ranging from 1 to 3 with 0.5 increments. For a given inter-marker region centered at position *p*, we then calculate *F*_*assoc*_(*β*), the number of associations that cross position *p *(i.e. associations that would be destroyed by a breakpoint).

A logistic regression was first applied on the data with inter-maker distance as unique predictive feature. Residuals obtained from the regression were then used to train multiple linear regression predictor. Using the residuals allows the capture of information that is not related to this inter-marker distance.

The 29 features are composed of four types of markers (coding metatheria, non-coding metatheria, coding amniote and non coding amniote) analyzed with 6 different *α *values and 5 different *β *values representing putative associations between a non-coding conserved region and a gene. The 29 features were first tested separately as single predictors and their effect on the prediction assessed with the t-value associated with the linear predictor output. Then, features were selected using a forward selection method. Each addition of a new feature was selected using the highest specificity value for a given sensitivity of 0.75. Sensitivity and specificity were calculated using the number of base pairs covered and not the number of inter-marker distance. We then undertook a four-fold cross validation on the oppossum genome and the predictor was finally applied on the human genome where further functional analysis is undertaken. It should be stated that we tried to add interactions between features but this didn't bring much improvement.

### Additional datasets

Gene desert are defined as gene-free regions spanning more than 1 Mb, based on the Ensembl gene annotation version 49. Only genes labeled as "known" were used. All regions with more the 1/3 of non-sequenced base pairs were removed from the dataset.

GNF Expression Atlas 2 [22] allows classifying genes depending on their expression in the 79 human organs and tissues covered by the Atlas. We considered that a gene is expressed in a given tissue if its MAS5 normalized expression level is > 400. We ported the GNF microarray probes to the Ensembl geneset using the Biomart tool [36] on the Ensembl web site.

## Authors' contributions

EM, KD and MB participated in the design of the study. EM carried out the development of the bioinformatics approach and analyzed data. EM and MB drafted the manuscript.

## Supplementary Material

Additional file 1 **Inter-marker region prediction scores**. A file containing the prediction score for each inter-marker region. Click here for file
